# Analysis of a CRDI diesel engine powered by ternary fuel blends (diesel, biodiesel, and pentanol) doped with alumina nano-additives

**DOI:** 10.1038/s41598-024-64929-1

**Published:** 2024-07-14

**Authors:** Mark Sudarsanam, Jayaraman Jayaprabakar, J. Aravind Kumar, Praveenkumar T R, M. Rajasimman

**Affiliations:** 1 Department of Mechanical Engineering, Sathyabama Institute of Science and Technology, Chennai, 600119 India; 2 Department of Mechanical Engineering, VEMU Institute of Technology, Chittoor, 517112 India; 3 Department of Energy and Environmental Engineering, Saveetha School of Engineering, SIMATS, Saveetha University, Chennai, 602105 Tamilnadu India; 4 Department of Construction Technology and Management, Wollega University, Nekemte, Ethiopia; 5 Department of Civil Engineering, Graphic Era Deemed to be University, Dehradun, India; 6 Department of Chemical Engineering, Annamalai University, Chidambaram, Tamilnadu India

**Keywords:** Diesel engine, CRDI, Emissions, Performance, Ternary fuel, Waste cooking oil, Biological techniques, Engineering

## Abstract

Environmental constraints associated with fossil fuels have driven researchers to find a novel, potential and environmentally benign alternative fuel. Biodiesel, vegetable oil, and alcohol have gained rapid momentum thanks to their renewable nature and comparable energy contents in recent years. Accordingly, a Ternary fuel blend is prepared comprising three fuels namely diesel, biodiesel, and pentanol. Waste cooking oil was identified as the source for biodiesel and Pentanol was chosen among various alcohol alternatives due to improved energy density, reduced toxicity. These are endorsed to the enhancement in surface area-volume ratio of nano additives which boosts the catalytic combustion activity and also causing lesser fuel to take part in combustion for maintaining a constant engine speed. The experimentation is done with ternaryfuel blends with varying pentanol and biodiesel concentrations of diesel, biodiesel and pentanol). Upon experimentation, it was observed that, ternary fuel blend ‘TF’ comprising 70% diesel, 20% biodiesel and 10% pentanol, yielded best performance and was used for doping of Alumina oxide (Al_2_O_3_) nano additives. The Al_2_O_3_ nanoparticles were doped with ternary blends at fractions of 10 ppm, 20 ppm, and 30 ppm. It was observed that 20 ppm Al_2_O_3_ nanoparticle blended TF blend improved BTE and lowered BSFC by about 12.01% and 22.57% respectively. The performance tremendously along with lowered the CO emission by 49.21%, HC emission by 18.91% and smoke opacity by 9.02%.

## Introduction

Increasing environmental concerns due to pollution caused by the industrial sector, agricultural sector, transportation sector and power sector, and steadily increasing fossil fuel prices due to its drop-in availability is the common problem faced presently by all developed and developing nations^1,2^. Progressively, there is steadily increase in population and their requirement of power, energy, and vehicles altogether stimulates the quench towards the search for potential alternative fuel. Conventional diesel combustion gases were estimated to emit 67% N_2_, 12% CO_2_, 11% H2O and 9% O_2_. Among various pollutants, Hydrocarbon (HC), Oxides of nitrogen (NOx), Carbon monoxide (CO) and smoke were the four major pollutants that cause damage to environment and human health, such as asphyxiation, impaired concentration, and retarded reflexes as a result of mixing of pollutants in hemoglobin. Higher pollutant exposure such as NOx emissions can even lead to cancer as the NOx emissions are carcinogens in nature. The depletion of worldwide fossil fuel reserves and escalating environmental concerns have propelled researchers to seek alternative, cleaner sources of energy, such as biodiesel. Diesel fuel additives, including organic compounds, present a viable solution to enhance the chemical and physical properties of in-cylinder charged fuel within vehicles^3^. Conventional combustion methods employed in Direct Injection (DI) diesel engines often result in high levels of emissions. Hence, there's a pressing need to develop new combustion modes that yield lower combustion temperatures and reduced emissions. The primary focus of this research is to investigate combustion performance and emission characteristics of Premixed Charge Compression Ignition (PCCI) in DI engines using blends of biodiesel and commercial diesel fuel. This aims to mitigate reactivity in blends of waste cooking oil and fossil diesel fuel mixtures^4^.

Biodiesel sourced from waste cooking oils has gained rapid momentum in recent years as they tend to emit reduced toxic emissions compared to conventional mineral diesel fuel. Even the first diesel engine demonstrated by Rudolf diesel was made to run by peanut-based biodiesel. However, long-term usage of biodiesel in engines can post certain drawbacks such as scuffing of piston rings, carbon deposits near values and lower thermal efficiency^5^. Also, higher viscosity biodiesel affects the atomisation and pumping characteristics. Alcohols can have blended with diesel fuel at lower concentrations. They are used as alternative fuel owing to reduced calorific value content and higher latent heat of the mixture. Also, alcohol blending in base fuel reduces NOx emissions significantly due to occurrence of low temperature combustion^6^. For homogenisation, biodiesel-alcohol blends and diesel-alcohol blends require specific stabilisers such as Span 80, Tween 20, CTAB. These stabilisers are pretty costlier, and hence the overall fuel cost shoots up higher. Therefore, several research works were done to overcome the usage of stabilisers, which paved way to open a new field of research, known as ternary blends. Even though binary fuels have several advantages and can be utilised directly in diesel engines, some problems such as mixture stratification, soot formation, reduced cetane number etc. were noticed which along with higher cost stabilisers lead to the usage of biodiesel in binary blend. Biodiesel addition (10–15%) in diesel-alcohol subsequently lowered the mixture stratification, opening a new pathway called ternary fuel blend^7^. Thereby, the fuel cost comes lesser than mineral diesel fuel. Hence, preparing a ternary fuel blend of biodiesel, diesel, and alcohol can be a potential alternative feedstock for existing CRDI diesel engine.

Rocha et al.^8^ analyzed ternary blends of biodiesel, ethanol and diesel fuel in different proportions in a diesel engine. The different proportions of base fuel include 5% of biodiesel from soybean oil in diesel, 99% pure anhydrous ethanol and hydrous ethanol with 7% of water. Five different volume fraction of biodiesel (5%, 10%, 20%, 60% and 100%) and four different volume fraction of ethanol (0%, 5%, 8% and 15%) were employed for the study. From the resulting outcome, various conclusions were drawn. NOx increased with more biodiesel content in a blend and also CO emission decreased. Changing ethanol concentration played an important role in result output. A clear reduction in NOx and Carbon dioxide (CO_2_) was observed. Cetane number of hydrous ethanol decreased than anhydrous ethanol-blended. Nour et al.^9^ tested an experimental CI engine using ternary fuel blends of pentanol, ethanol, diesel (Pe10E10D80) and octanol, ethanol and diesel (Oc10E10D80). Properties of fuel were found initially and the thermogravimetric analysis of fuels was performed. The experiment was performed on diesel engine at different operating condition. Result showed that peak and in-cylinder pressure for Pe10E10D80 and Oc10E10D80 were lower than base fuel diesel. Combustion duration and ignition delay period increased, enhancement in diffusion combustion zone and diminishing premixed composition phase is noted for HRR. BSFC is lower for Oc10E10D80 than diesel but is higher for Pe10E10D80 than diesel. BTE for Oc10E10D80 is higher than diesel, for Pe10E10D80 it is lower. Emission such as CO_2_, CO, NOx and smoke reduced by 56%, 83%, 33% and 73% respectively.

Teoh et al.^10^ conducted an experiment in a diesel engine to check emission, performance and combustion characteristics of coconut biodiesel-diesel blend in common rail turbocharger diesel engine operating at different torque. Blends B20 (20% biodiesel + 80% diesel), B20E5 (20% biodiesel + 5% bioethanol) and B20E10 (20% biodiesel + 10% bioethanol) blends were used for testing. Results showed that BTE improved better for bioethanol blend for the cost of BSFC. Improvements in NOx were found. Reduction in smoke and CO was found. HRR and peak in-cylinder pressure to be high for B20E10. Thus the present study showed that B20E10 blend exhibited better improvement than other in a diesel engine. Ghadikolaei et al.^11^ investigated a four stroke diesel engine using various alternative fuels with five loads. Oxygen content in all blends of 5%, close carbon-hydrogen content and low heating value were also maintained. Outcome results clearly revealed that reduction in emissions such as CO_2_, CO, HC, NOx and PM is observed for all test fuel blends except mineral diesel. DBM showed low BSFC, high BTE and lowest emissions such as HC, CO_2_, CO, NOx and PM next to DBPr. Highest peak in HRR and Pθ is observed for all fuel except DB.

Raman et al.^12^ done a study on four stroke diesel engine operating with rapeseed oil biodiesel as testing fuel. Transesterification was done to convert raw rapeseed oil into biodiesel. Fuel properties of methyl ester of rapeseed oil were found to cope well with engine hardware. Hence engine modification is not required. Experiment was done in a 5.95 kW single-cylinder, four stoke diesel engine. Various engine characteristics such as BSEC, BSFC, BTE, exhaust gas temperature and HRR, in-cylinder pressure and delay period was evaluated. Emission such as HC, NOx, CO and smoke emission of engine measured for all tested fuel. Experiment was conducted and resulting outcome showed better results for B25 (25% biodiesel + 75% diesel) without engine modification. Nandagopal et al.^13^ investigated a ternary fuel blend (biodiesel, diesel and decanol) on a single-cylinder DI diesel engine. Decanol was chosen as an ignition improver. Various concentrations of decanol such as 10%, 20%, 30% and 40% by volume were blended with diesel and CalophyllumInophyllum biodiesel. Outcome of the study clearly revealed that experimental engine operation with ternary blend showed an increase in BTE with an increase in decanol concentration and BSFC reduced. An increase in alcohol concentration showed a rise in NOx emission. 40% decanol blend showed highest peak pressure and 10% decanol showed lowest peak pressure. HRR and CHRR decreased at combustion end phase. Finally, it was concluded that a 40% blend of decanol showed optimised results without any engine modification.

Ashok et al.^14^ experimented with a four stroke diesel engine using CalophyllumInophyllum blend with various percentages of isobutanol as additive fuel. Five blends were prepared initially D80IB20 (80% diesel + 20% isobutanol), B80IB20 (80% biodiesel + 20% isobutanol), three ternary blends (biodiesel-diesel-isobutanol) of 10%, 15% and 20% concentration of isobutanol. Fuel properties were found and experiments were conducted. BTE improved by 3.19% and better BSFC for 10% iso-butanol blend were observed when compared to biodiesel. CO emission reduced by about 13–59% for all blends than diesel fuel with a slight increase in HC emission. NOx emission reduction by about 8.16% is observed for all isobutanol added fuel blends than biodiesel but higher than diesel. Isobutanol can be used as a feasible additive to replace partial diesel in engine applications. Anchupogu et al.^15^ studied the influence of Al_2_O_3_ addition in CalophyllumInophyllum Biodiesel (CIB) as a testing fuel in a diesel engine. Al_2_O_3_ nanoparticle with 40 ppm was dispersed in CIB. CIB20 (20% CIB + 80% diesel) is doped with 40 ppm Al_2_O_3_ by ultra-sonication technique. CIB2OANP40 (CIB20 + 40 ppm Al_2_O_3_) and CIBI20ANP40 + 20% EGR (20% of Exhaust Gas Recirculation) blends were also tested. Results showed that BTE of CIB20ANP40 increased by 5.04 and 7.71% when compared with CIB20 blend and CIB20ANP40 + 20% EGR blend fuel. Al_2_O_3_ addition reduced HC and CO emission than CIBI20. NOx for CIB20ANP40 + 20% EGR reduced by 36.84%, 31.53% and 17.67% than CIB20, CIB20ANP40 and CIB20 + 20% EGR fuel blends, respectively. Khatri et al.^16^ experimental investigated ZnO nanoparticle as an additive in diesel fuel. ZnO nanoparticle doped with diesel from 5 to 25 mg. Magnetic and ultrasonic process was used to mix ZnO in diesel fuel. Compression ratio of 18 is maintained with increasing engine load of 2 to 12 kW have experimented. Thermal efficiency improved by 15.58% and a reduction of 11.11% in BSFC for ZnO20DF60 (20 mg ZnO + 20 mg CTAB surfactant + 600 ml diesel fuel) were noted than diesel. Emission wise HC, CO, CO_2_, NOx and PM were reduced by 78.78%, 58.93%, 41.85%, 57.46% and 42.51% respectively. ZnO addition in diesel proved to be a better alternative fuel for less emission, and thus it is considered as an eco-friendly fuel. In response to the escalating global focus on sustainability, a groundbreaking, eco-friendly material has emerged as a high-performance substitute for petrochemical-based materials. The drive towards miniaturization and seamless integration has catalyzed a robust technical revolution, particularly in the realm of wearable electronics. Consequently, there's a burgeoning demand for all-solid-state flexible energy storage devices^17–19^.

Pentanol was chosen among various alcohol alternatives due to improved energy density, reduced toxicity, easier handling, transportation and engine storage. Experiments were performed at 3.7 kW, 4-stroke CRDI diesel engine for analyzing the emission and performance parameters of conventional diesel fuel and ternary blends. The ternary fuel blends were prepared with varying pentanol concentration and the best blend is chosen based on improved performance and emissions. Further modifications were done by using Aluminium oxide or alumina (Al_2_O_3_) nano additives with different doping concentrations of 10 to 30 ppm owing to their improved catalytic properties and higher surface area-volume ratio. The best concentration among 10, 20 and 30 ppm were further subjected to subsequent experimentation for bringing out best.

## Materials and methods

**Figure 1:**
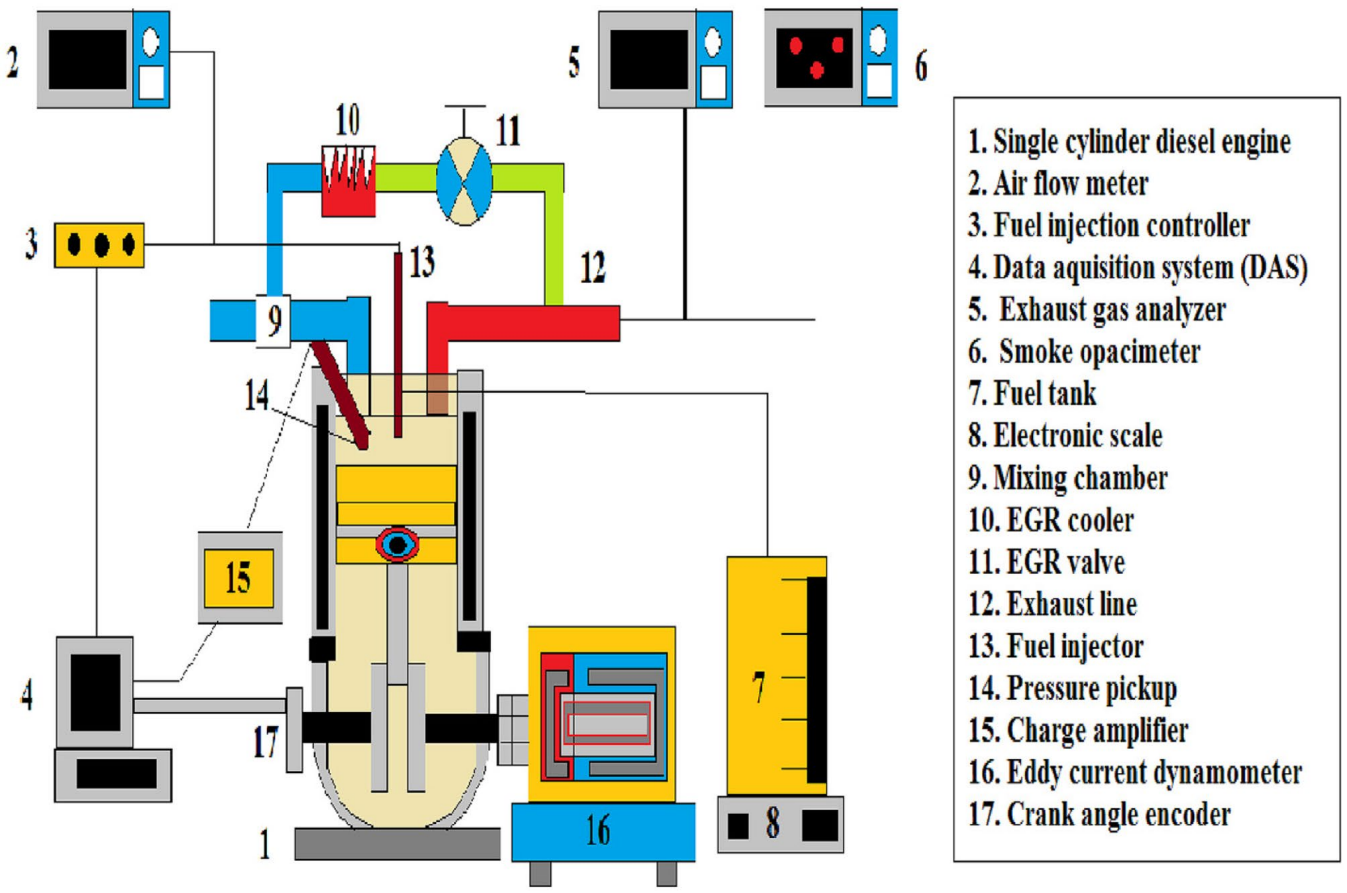
Schematic view of experimental setup^20^.

The present experimental research, a Kirloskar make, four stroke, single cylinder, CRDI diesel engine is employed. This engine is coupled with series of instruments to bring out the fuel performance and the test engine with variable loads from 0 to 100% at intervals of 25%. To track RPM and load, the CRDI engine is paired with an Eddy current dynamometer. Cylinder pressure and temperature were monitored using pressure sensors and thermocouples, respectively. Additionally, exhaust gas emissions were analyzed using an exhaust gas analyzer. It is intended to perform the fuel alterations that is doping of nano additives in ternary blend. The photographic view of experimentation setup is displayed in Fig. 1. The engine develops around 3.7 kW rated output power at 1500 rpm. Bore and stroke of the engine are 87.5 mm and 110 mm with a compression ratio of 17.5:1. The engine manufacturer specified standard injection timing and standard injection pressure as 23° bTDC and 600 bar, respectively. The specifications of test engine employed for experimentation are given in Table 1.

**Table 1:** Engine specifications.

Particulars	Specifications
Type	Kirloskar—4S CRDI diesel engine
No. of cylinder	Single-cylinder
Compression ratio	17.5:1
Standard injection timing	23°bTDC
Bore length	87.5 mm
Stroke length	110 mm
Power	3.7 kW
Injection pressure	600 bar
Speed	1500 rpm

The comparison of various physico-chemical properties of diesel fuel and Waste Cooking Oil biodiesel (WCO) is indicated in Table 2. Main properties of neat biodiesel have not suitable for better emission and performance characteristics since the diesel engine is designed to operate diesel fuel alone. Hence, a novel ternary blend fuel has been developed for existing diesel engine. A novel ternary blend of diesel, biodiesel, and pentanol is blended and stirred using a magnetic stirrer for 2 h. Further, Al_2_O_3_nano additives were added in a concentration of 10 ppm, 20 ppm, and 30 ppm named as TF + A10, TF + A20 and TF + A30 respectively and the important test fuel properties such as viscosity, density, calorific value, flash point and cetane number were determined as per ASTM norms. The doping of metal oxides has gained significant traction in recent years due to their wide-ranging applications in both materials and thermal approaches. The addition of trace quantities of metal oxides inevitably enhances the optical, structural, magnetic, and thermal properties of materials. This enhancement is particularly notable in nano additives, which boast a high surface area to volume ratio, thus significantly improving combustion efficiency. Among the plethora of metal oxides, Al_2_O_3_ nanoparticles stand out for their effectiveness in simultaneously enhancing performance and reducing emissions. Despite the availability of various technologies for synthesizing nanoparticles, such as the precipitation method and chemical vapor deposition, the traditional sol–gel combustion method remains widely employed. This method typically involves four key steps: solution preparation, gel formation, particle growth, and particle agglomeration. Table 2 displays the main physio-chemical properties of test fuels. The properties of Al_2_O_3_ nanoparticles were given in Table 3.

**Table 2:** Physio-chemical properties of test fuels.

Fuel properties	D100	B100	B50	TF	TF + A10	TF + A20	TF + A30	ASTM method
Density (kg/m^3^)	840	874	859	834	831	830	827	D1298
Kinematic viscosity at 35 °C, cSt	2.84	4.34	3.27	3.71	3.58	3.52	3.23	D445
Calorific value (kJ/kg)	44,700	42,673	43,146	44,456	44,652	45,017	44,841	D240
Flash point (°C)	68	130	50.25	73.9	75	78	77	D93
Cetane number	48	52.7	98.7	46.45	46.7	46.8	46.5	D613

**Table 3:** Al_2_O_3_ nanoparticle properties.

Particulars	Values
Bulk density (g/cm^3^)	0.18
Crystal structure	γ
Appearance, colour	White
Purity (%)	94.99
Surface area, m^2^/g	225.44
Dislocation density (δ), line^2^/m^2^	0.4 × 1014
Average particle size, nm	27–30

## Results and discussion

The experimentation is done with ternary blends with varying pentanol concentrations. Upon experimentation, it was observed that, ternary fuel blend ‘TF’ comprising 70% diesel, 20% biodiesel and 10% pentanol, yielded best performance and was used for doping of Alumina oxide (Al_2_O_3_) nano additives. The Al_2_O_3_ nanoparticles were doped with ternary blends at fractions of 10 ppm, 20 ppm, and 30 ppm. The ternary blends include TF (70% diesel + 20% biodiesel + 10% pentanol), TF + A10 (TF + 10 ppm Al_2_O_3_), TF + A20 (TF + 20 ppm Al_2_O_3_) and TF + A30 (TF + 30 ppm Al_2_O_3_) respectively. In this research, alumina nanoparticles were employed with three concentrations namely 10 ppm, 20 ppm, and 30 ppm and their performance, combustion, and emission characteristics were studied to find which concentration is effective.

### Performance characteristics

**Figure 2:**
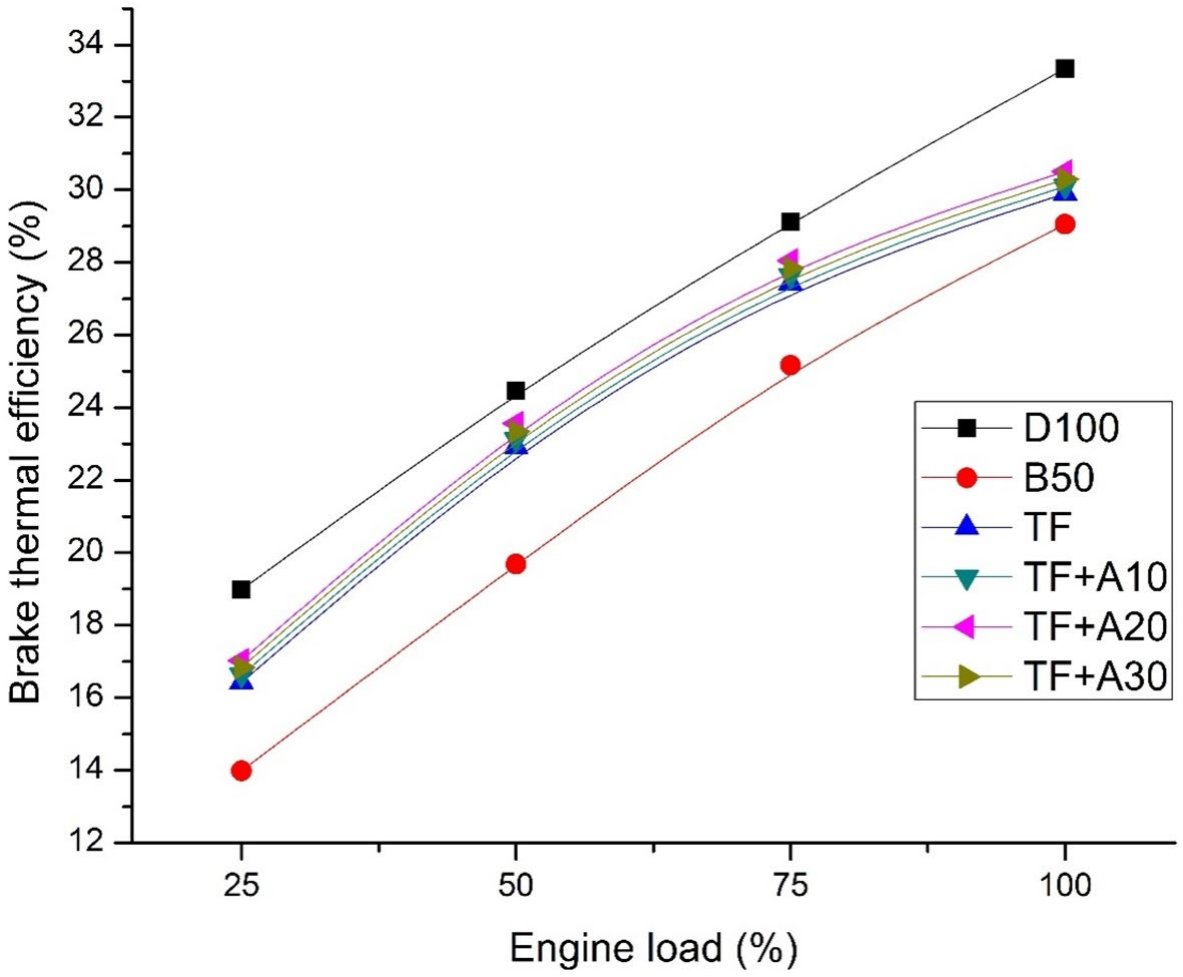
BTE vs engine load.

Figure 2 displays the fluctuation of BTE for diesel, biodiesel blend and ternary blends doped with 10 ppm, 20 ppm and 30 ppm alumina nanoparticles with respect to load. It is inferred that B50 blend shows lowest BTE blends during all engine loads which can be attributed to higher density and viscosity of the blend. In comparison to D100, the viscosity of B50 is higher by about 15.14% thus resulting in lowest BTE of 29.05% at 100% load representing in adequate combustion. However, the presence of pentanolin the ternary fuel tends to improve the BTE significantly owing to the O_2_content in the blend followed by enhanced combustion efficiency. However, the BTE of ternary blends were lower than that of D100. Addition of alumina nano additives in ternary blends enhances the BTE remarkably. From the fuel property Table 2, it is evident that, addition of Al_2_O_3_ nanoparticles in TF blends causes improvement in calorific value and a noticeable drop in kinematic viscosity of mixture. The BTE of nano additives doped TF were increased by about 0.67%, 2.01%, and 1.34% with additive concentrations of 10, 20 and 30 ppm respectively in comparison with TF. This could be perhaps, owing to the catalytic combustion process caused by the Al_2_O_3_ nanoparticles, which promotes micro-combustion of fuel droplets thus enhancing the rate of evaporation and lowering the physical delay which altogether improves the combustion and BTE subsequently. Addition of nanoparticle causes BTE improvement also owing to higher surface area-volume ratio of Al_2_O_3_ nanoparticles which causes catalytic reactivity. Among different dosage concentration, the 20 ppm alumina concentrations (TF + A20) blend showed better improvement in BTE when compared with 10 ppm and 30 ppm concentrations. These results are in good coherence with several research findings^21,22^.

**Figure 3:**
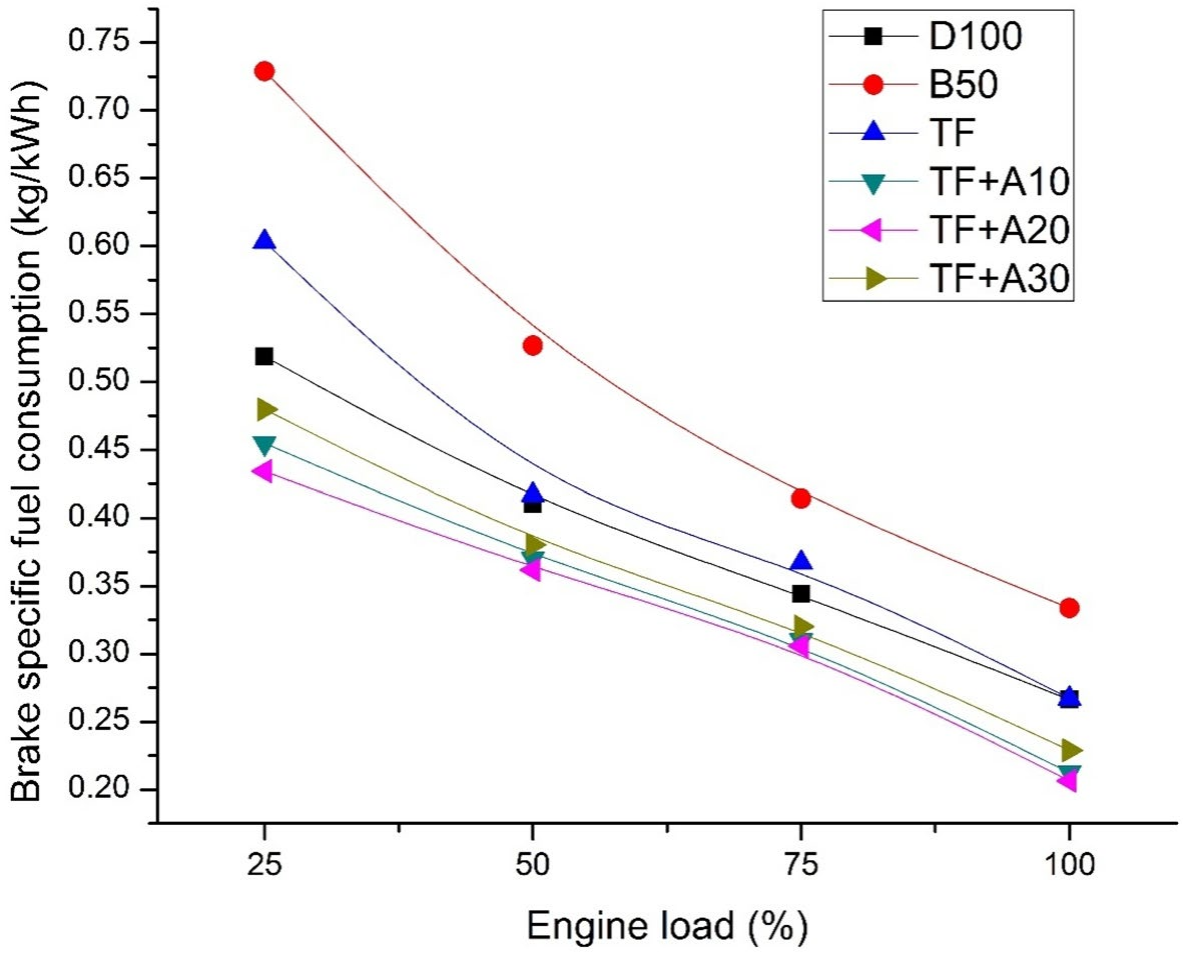
BSFC vs engine load.

Figure 3 shows the fluctuation in BSFC for diesel, biodiesel blend and ternary blends doped with 10, 20and 30 ppm Al_2_O_3_nanoparticles with respect to load. It is clear that B50 blend displays highest fuel consumption than the other blends. The heating value of B50 is lesser than mineral diesel fuel by about 2.78% which is the main reason for BSFC variation between them. Moreover, this higher viscosity of B50 causes poor atomisation and vaporisation tendency thereby increasing the BSFC trend. TF blend has lowered BSFC than B50 by about 40.5%, 28.46%, 20.33%, and 25.38% at engine loads of 25, 50, 75, and 100% respectively. It can be validated due to the influence of pentanol resulting in improved combustion efficiency and less quantum of fuel entering the combustion chamber for maintaining the engine speed constant in comparison to B50 blend which is lower calorific value fuel. Addition of Al_2_O_3_ nanoparticles tends to lower the BSFC considerably. Lowest BSFC is observed for TF + A20 through all loads. At 100% engine load, the BSFC of TF + A20 is 206 g/kWh which is 22.57% lower than diesel. It can be attributed to the improved surface area-volume ratio of Al_2_O_3_ nanoparticles paving way to lowering physical delay and enhanced cetane characteristics thereby lowering the BSFC subsequently. Among different dosage concentrations of Al_2_O_3_ in TF, it is found that TF + A20 blend resulted in lowest BSFC owing to huge surplus energy released by mixture during Al_2_O_3_ combustion. Also, the improvement in calorific value of TF + A20 is compared to other concentrations of 10 ppm and 30 ppm results in an improved rate of evaporation, reduced physical delay period, lessened ignition delay along with elongated spray penetration causing better combustion efficiency of TF + A20 followed by lower BSFC. Similar reductions in BSFC with Al_2_O_3_nano additives were also quoted by several researchers^21,23^.

### Emission characteristics

**Figure 4:**
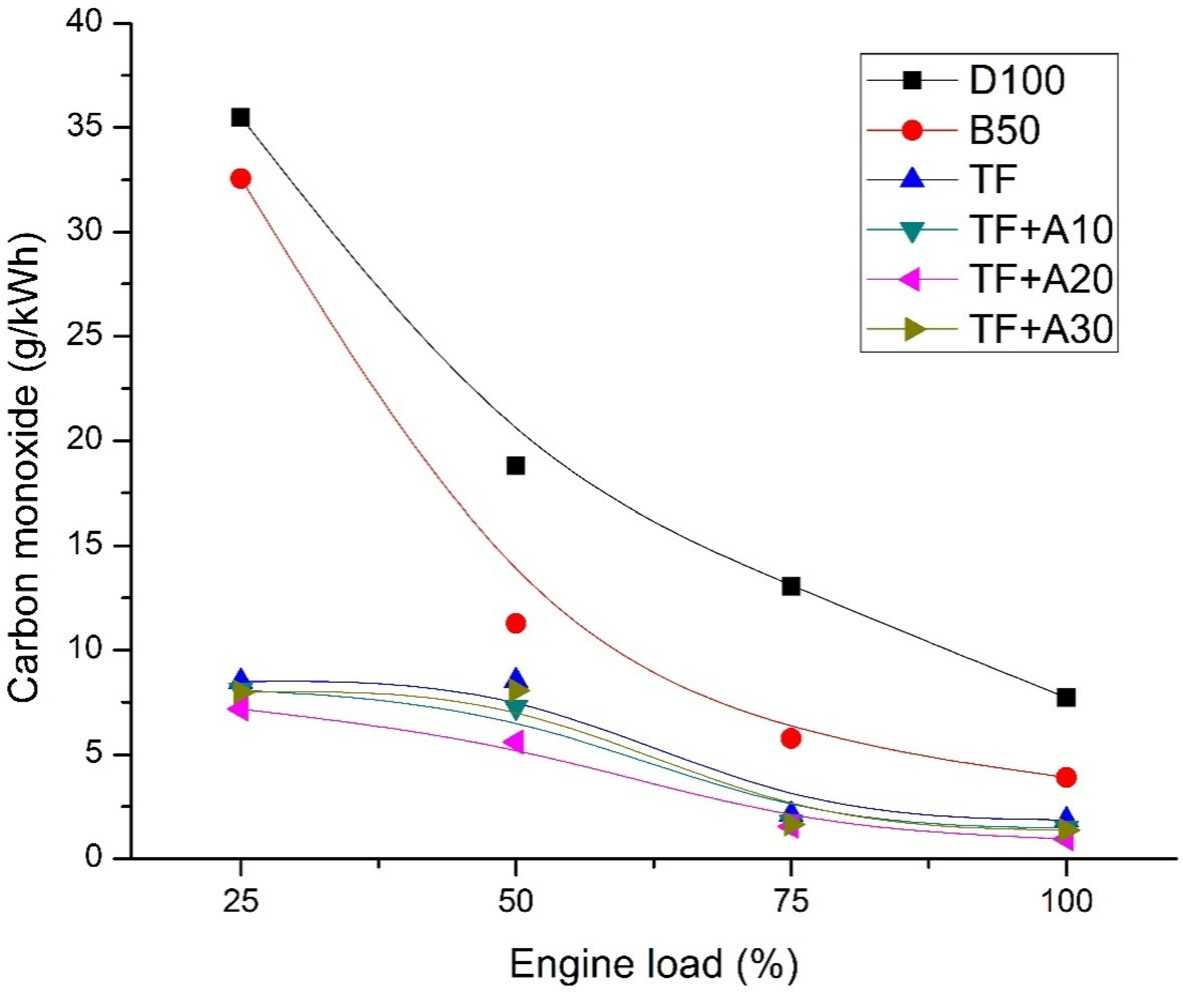
CO emission vs engine load.

Figure 4 displays the variation of CO emission for diesel, biodiesel blend and ternary blends doped with alumina nanoparticles with respect to engine load. It can be observed that, the highest CO emission is recorded for D100 through all loads. B50 blend, in comparison with D100 has lowered CO emission by about 8.24%, 39.97%, 55.79% and 49.52% at engine loads of 25, 50, 75 and 100% respectively. These can be owing to the presence of excess O_2_ atoms in B50 blend which stimulates the conversion of CO to CO_2_ molecule. Ternary blends found to produce lower CO emissions than D100 and B50 blend which can be attributed to the presence of pentanol which functions as a combustion intensifier, thereby inducing rapid combustion rate followed by lessened CO emissions. Addition of Al_2_O_3_ nanoparticle TF further reduces the CO emissions. At 100% engine load condition, CO emission of TF + A10, TF + A20 and TF + A30 were lower than TF by about 20.53%, 49.21%, and 25.94%. This could be due to Al_2_O_3_nano additives acts as an O_2_ buffering agent and O_2_ donating catalyst for oxidising the CO molecule. Al_2_O_3_nano additives tend to reduce the ignition delay period followed by complete combustion^24^. Similar studies with Al_2_O_3_ nanoparticles addition lowering the CO emissions were shown by several researchers^25,26^.

**Figure 5:**
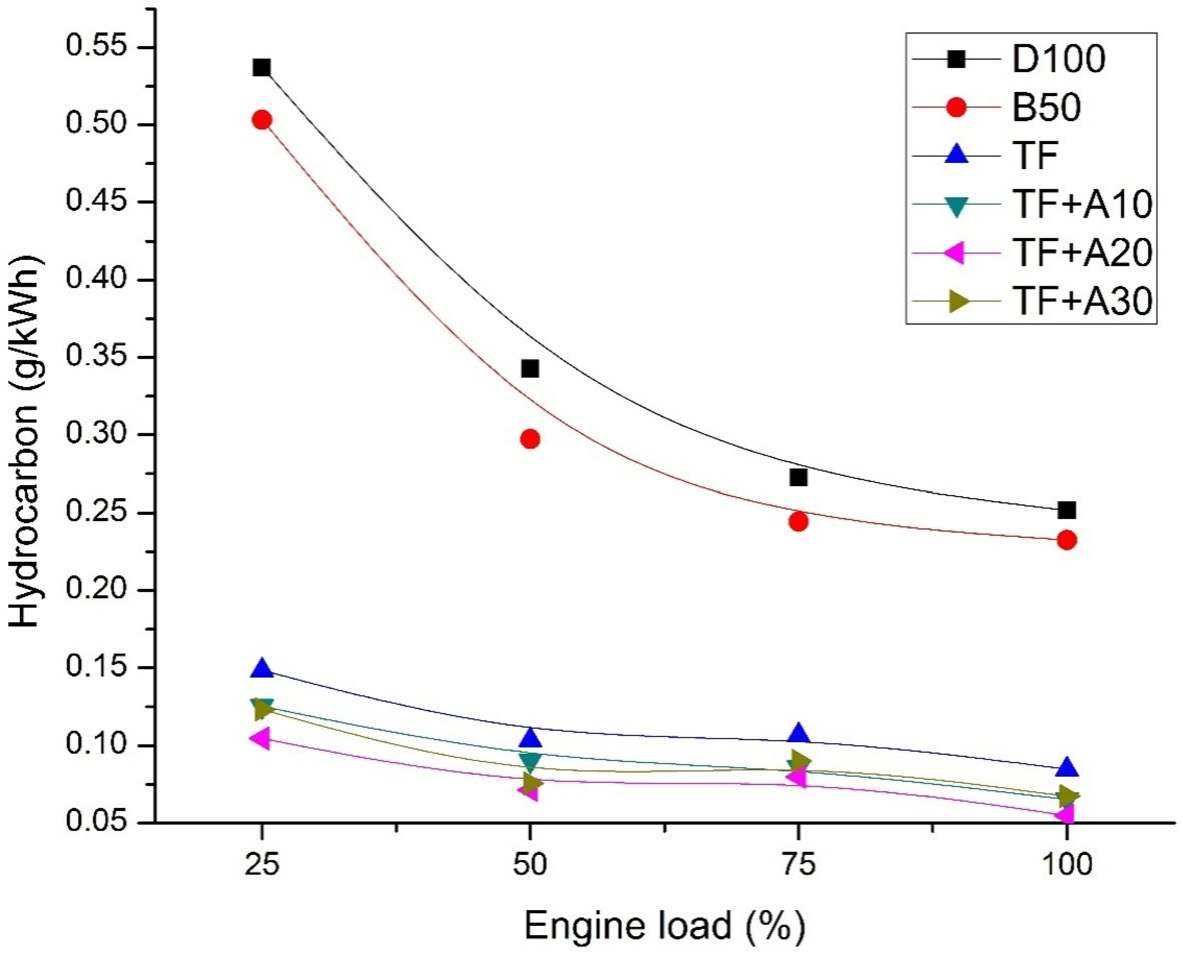
HC emission vs engine load.

Figure 5 displays the variation of HC emission for diesel, biodiesel blend and ternary blends doped with alumina nanoparticles with respect to load. It can be perceived that, D100 displays highest unburned HC emissions in comparison to ternary blends and ternary blends doped with nano additives. With increments in load, HC emissions were found to increase due to more fuel quantum taking part in combustion. Ternary blends exhibits lowered HC emissions in comparison with other test blends owing to the presence of pentanol which contributes instant O_2_ throughout the course of combustion thus oxidising the soot-precursors resulting in complete combustion and lowered emissions of hydrocarbon. At 100% engine load, HC emissions of TF were lesser than D100 and B50 by about 66.31% and 63.53% respectively. Appending Al_2_O_3_ addition in TF resulted in lowered HC for all the concentrations in compared with mineral diesel and biodiesel blends. This is because of the activation energy of Al_2_O_3_ particles leading to ignite the carbon sediments inside the engine, specifically at the layers of quenching. At full load, the HC emission of TF + A10, TF + A20, TF + A30 is lower than TF by about 23.11%, 18.91%, and 3.15%, respectively. The oxygen presence in Al_2_O_3_nano additives reduced the nano additives, reduced the fuel mixture’s viscosity and improved surface area of the mixture which completely results in an enhanced heat transfer rate. Thus, TF + A20 ensued in lowest hydrocarbon emissions when compared with other concentrations. These are good accordance with research findings of^21,27^.

**Figure 6:**
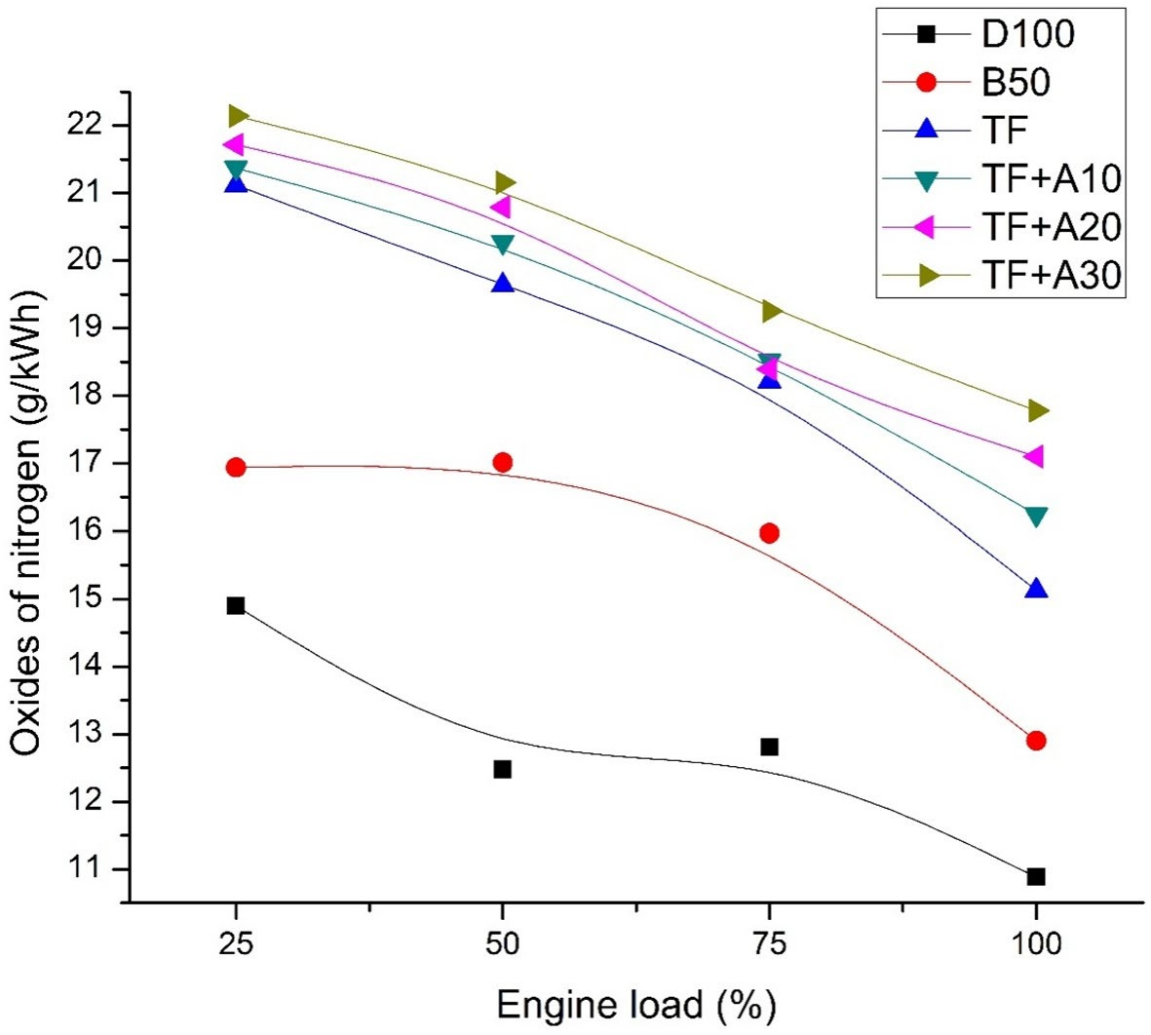
NOx emission vs engine load.

Figure 6 indicates the variation of NOx emissions for diesel, biodiesel blend and ternary blends doped with alumina nanoparticles with respect to engine load. It can be observed all the oxygenate blends exhibit higher NOx emissions due to the existence of oxygen molecule which could possibly result in improved cylinder temperatures that favour NOx formation chemistry. The NOx emissions of TF are higher than D100 and B50D50 by about 39.03% and 17.26% respectively at full load. Also, the additions of Al_2_O_3_ nanoparticles in TF blend have found to improve the NOx emission subsequently. At full load, the NOx emissions of TF + A10, TF + A20, and TF + A30 were higher than TF by about 7.36%, 13.05% and 17.77%, respectively. This increase in perhaps, attributed to the oxygen buffering tendency of nanoparticles which along with higher in-cylinder temperatures promoting the O_2_ and N_2_ mixing followed by higher NOx emission. However, 20 ppm concentration of Al_2_O_3_nano additives caused slightly lowered NOx emission and were in par with TF which could be attributed to reduction reactions at optimum concentration. These are also in good agreement with the research findings were 20 ppm concentration of nano additives tends to lower the NOx formation. With increasing nanoparticle concentration, the NOx emissions are found to decrease in the similar literatures^24,28^.

**Figure 7:**
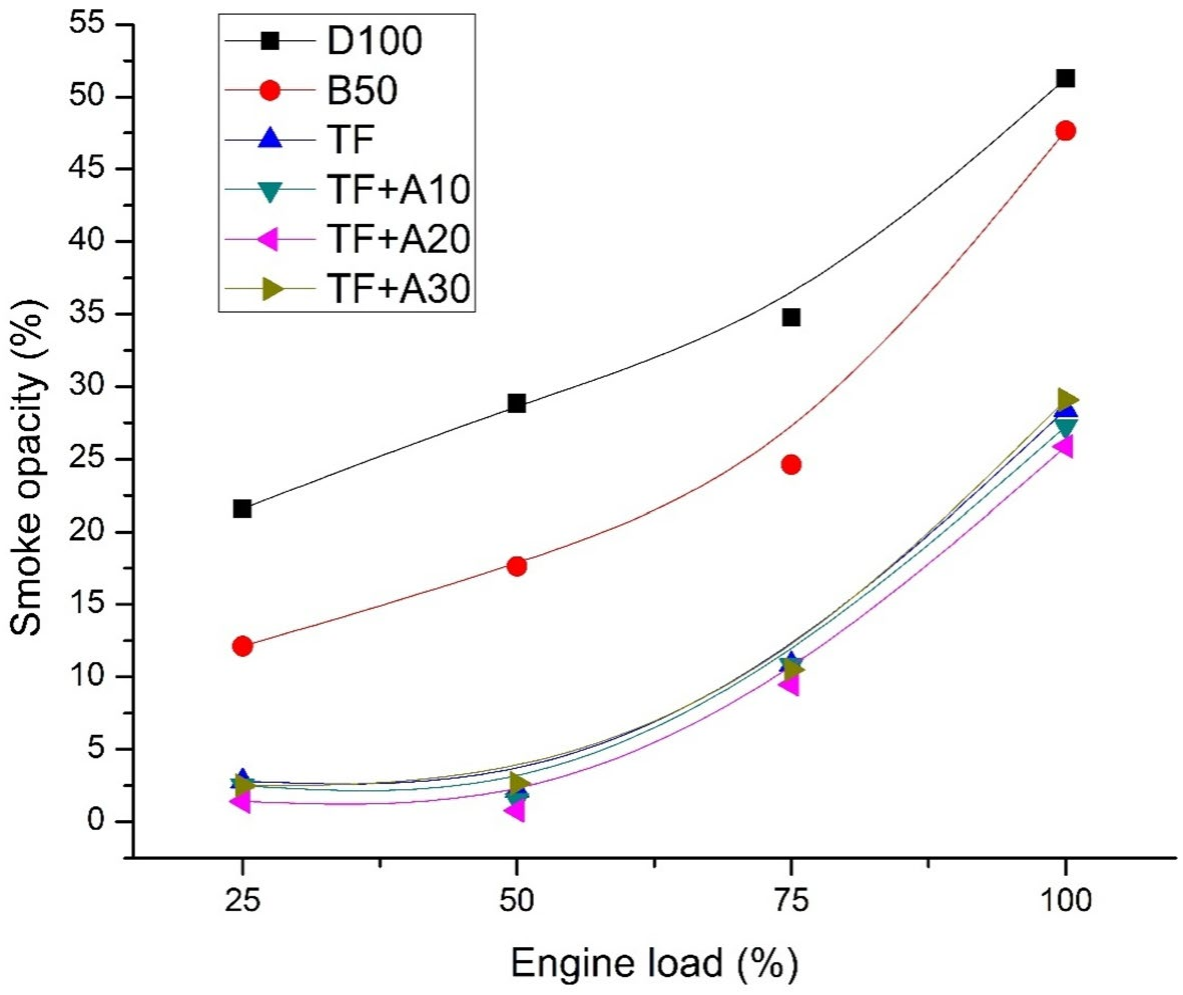
Smoke opacity vs engine load.

Figure 7 displays the variation of smoke opacity for diesel, biodiesel blend and ternary blends doped with alumina nanoparticles with respect to load. It is inferred that D100 shows highest smoke emissions. Oxygenated blends lowered the smoke due to the presence of in-built oxygen content in their chemical structure paving way for complete combustion. In comparison with B50, smoke reduction for TF is enormous owing to fumigation of pentanol leading to better oxidation and increased combustion efficiency. Addition of Al_2_O_3_ nanoparticles in the TF blend found to further lower the smoke emissions. At 100% load, the smoke emission of TF + A10, TF + A20 are lower than TF by about 4.16% and 9.02% respectively. TF + A30 alone have 2.3% higher smoke than TF blend. Lowest smoke trend is observed for TF + A20 through all engine loads. It can be endorsed to the existence of an optimum concentration of nano additives causing improvement in evaporation, reduction in ignition delay and enhanced combustion efficiency. At lowered delay period, more fuel quantum is trapped inside the chamber before ignition thereby ensuing improved fuel–air mixing and reduce the smoke emissions. 20 ppm Al_2_O_3_ concentration showing a major drop in smoke opacity can also be due to optimum fuel viscosity and improved cetane number tendency of fuel influencing the fuel spray and droplet size characteristics thereby lower the smoke emissions subsequently. Lowered smoke emission with alumina additives was in common with findings of^27,29^.

## Conclusion

The experimentation was carried out using TF blend blended with Al_2_O_3_ nanoparticles at three different concentrations of 10, 20 and 30 ppm. Based on experimentation, the following can be concluded subsequently.

- Performance wise, addition of 20 ppm alumina nanoparticles in ternary blends have resulted in significant improvement in BTE and lowered BSFC by about 12.01% and 22.57% respectively when compared with mineral diesel.
- Emission wise, addition of 20 ppm alumina nanoparticles in ternary blends lowered the CO emission by 49.21%, HC emission by 18.91% and smoke opacity by 9.02%. However, NOx emissions of alumina nanoparticle blended TF blend resulted in 13.05% higher NOx than TF blend without additives.
- However, higher NOx emissions with nanoparticle addition are attributed to the O_2_ buffering capability of nanoparticles which along with high in-cylinder air temperature promotes the reaction of O_2_ and N_2_ to form NOx.
- However, this work still has future scopes and can be extended possibly. Fuel spray pattern analysis with a microscopic camera placed inside the combustion chamber for the ternary blends. Exhaust gas recirculation technique with the present optimisation condition and employing artificial neural network approach for precise optimisation of engine parameters.

## Data Availability

The datasets used and/or analysed during the current study available from the corresponding author on reasonable request.

## Abbreviations

B100: 100% Biodiesel
D100: 100% Diesel
TF: 70% Diesel + 20% biodiesel + 10% pentanol
B50: 50% Diesel + 50% biodiesel
Al_2_O_3_: Aluminium oxide or alumina
ASTM: American society of testing and measurement
BTE: Brake thermal efficiency
BSFC: Brake specific fuel consumption
NOx: Oxides of nitrogen
CO: Carbon monoxide
HC: Hydrocarbon
WCO: Waste cooking oil
TF + A10: 70% Diesel + 20% biodiesel + 10% pentanol doped with 10 ppm Al_2_O_3_ nanoparticle
TF + A20: 70% Diesel + 20% biodiesel + 10% pentanol doped with 20 ppm Al_2_O_3_ nanoparticle
TF + A30: 70% Diesel + 20% biodiesel + 10% pentanol doped with 30 ppm Al_2_O_3_ nanoparticle
